# Respiratory complex I with charge symmetry in the membrane arm pumps protons

**DOI:** 10.1073/pnas.2123090119

**Published:** 2022-06-27

**Authors:** Franziska Hoeser, Hannes Tausend, Sinja Götz, Daniel Wohlwend, Oliver Einsle, Stefan Günther, Thorsten Friedrich

**Affiliations:** ^a^Institute of Biochemistry, Faculty of Chemistry and Pharmacy, Albert-Ludwigs-University, 79104 Freiburg, Germany;; ^b^Institute of Pharmaceutical Sciences, Faculty of Chemistry and Pharmacy, Albert-Ludwigs-University, 79104 Freiburg, Germany

**Keywords:** biological energy conversion, respiratory chain, complex I, NADH dehydrogenase, proton translocation

## Abstract

Energy-converting NADH:ubiquinone oxidoreductase, respiratory complex I, is essential for cellular energy metabolism coupling NADH oxidation to proton translocation. The mechanism of proton translocation by complex I is still under debate. Its membrane arm contains an unusual central axis of polar and charged amino acid residues connecting the quinone binding site with the antiporter-type subunits NuoL, NuoM, and NuoN, proposed to catalyze proton translocation. Quinone chemistry probably causes conformational changes and electrostatic interactions that are propagated through these subunits by a conserved pattern of predominantly lysine, histidine, and glutamate residues. These conserved residues are thought to transfer protons along and across the membrane arm. The distinct charge distribution in the membrane arm is a prerequisite for proton translocation. Remarkably, the central subunit NuoM contains a conserved glutamate residue in a position that is taken by a lysine residue in the two other antiporter-type subunits. It was proposed that this charge asymmetry is essential for proton translocation, as it should enable NuoM to operate asynchronously with NuoL and NuoN. Accordingly, we exchanged the conserved glutamate in NuoM for a lysine residue, introducing charge symmetry in the membrane arm. The stably assembled variant pumps protons across the membrane, but with a diminished H^+^/e^−^ stoichiometry of 1.5. Thus, charge asymmetry is not essential for proton translocation by complex I, casting doubts on the suggestion of an asynchronous operation of NuoL, NuoM, and NuoN. Furthermore, our data emphasize the importance of a balanced charge distribution in the protein for directional proton transfer.

## Significance

Respiratory complex I is a central enzyme of cellular energy metabolism coupling quinone reduction with proton translocation. Its mechanism, especially concerning proton translocation, remains enigmatic. Three homologous subunits that contain a conserved pattern of charged and polar amino acid residues catalyze proton translocation. Strikingly, the central subunit NuoM contains a conserved glutamate residue at a position where conserved lysine residues are found in the other two subunits, resulting in a charge asymmetry discussed to be essential for proton translocation. We found that the respective glutamate to lysine mutation in *Escherichia coli* complex I lowers the amount of protons translocated per electron transferred by one-quarter. These data clarify the discussion about possible mechanisms of proton translocation by complex I.

The universal cellular energy currency adenosine triphosphate (ATP) is mainly produced by oxidative phosphorylation (OxPhos). OxPhos couples the activity of respiratory electron transfer chains with that of ATP synthase (1–3). The enzyme complexes of the respiratory chains use the redox energy obtained by the oxidation of the respective substrates to generate a protonmotive force (PMF) across a membrane. The PMF is used in turn by the ATP synthase to drive ATP synthesis (1, 2). Energy-converting NADH:ubiquinone oxidoreductase, respiratory complex I, is the main entry point for electrons from NADH into many electron transfer chains. Complex I couples the transfer of two electrons from NADH to a quinone (Q) with the translocation of four protons across the membrane (4–9). It has a two-part structure, consisting of a peripheral arm catalyzing electron transfer and a membrane arm conducting proton translocation (10). Thus, the complex performs proton-coupled electron transfer over a distance of more than 300 Å. The mitochondrial complex of mammals comprises 45 subunits, 14 of which represent the catalytic core. Homologs of these 14 subunits are found in all species that contain an energy-converting NADH:Q oxidoreductase. Seven of these core subunits are most hydrophobic, and are accordingly all located in the membrane arm of the complex. These subunits are mitochondrially encoded in eukaryotes hampering their genetic manipulation (11), although this is easily done in bacterial complex I, such as that of *Escherichia coli*. This enables the straightforward characterization of effects of mutations in the homologs of the mitochondrially encoded subunits on the stability, assembly, and catalytic activity of the variant complex (12–17). *E. coli* complex I comprises 13 subunits called NuoA to NuoN (from NADH:ubiquinone oxidoreductase) due to the fusion of the genes encoding subunit NuoCD. This complex represents a structural minimal model of an energy-converting NADH:Q oxidoreductase (18).

**Fig. 1.**
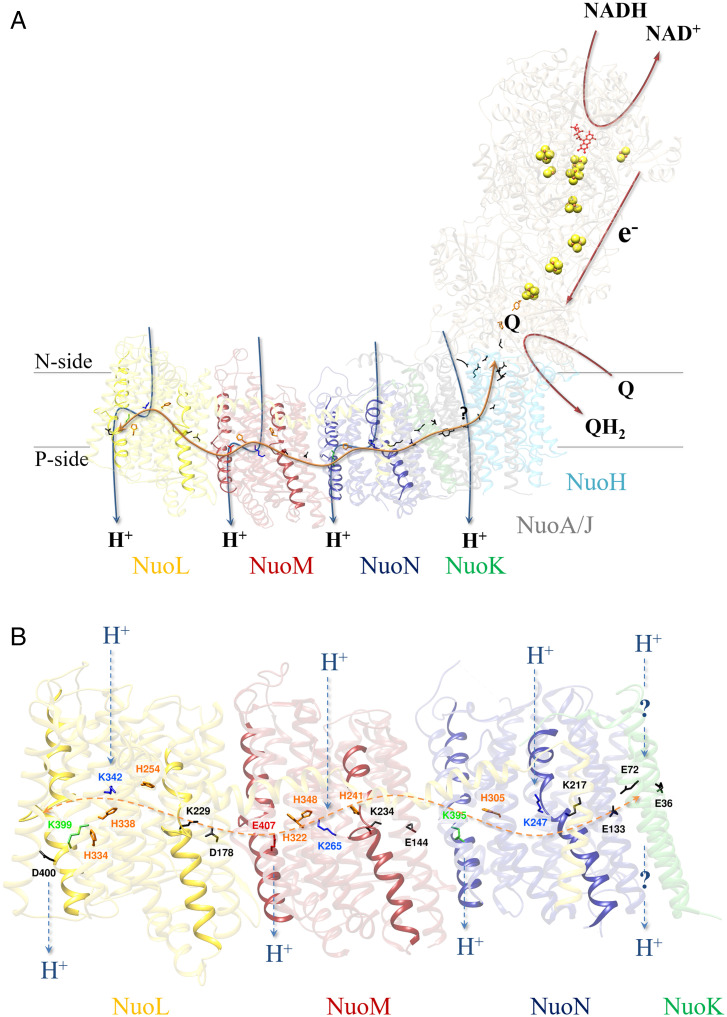
Structure of *E. coli* complex I. NuoL is shown in yellow, NuoM in red, NuoN in blue, and NuoK in green. Residues of interest are shown in stick and ball representation. The ion pairs are shown in black, the central lysine residues in blue, the distal lysine residues (NuoL and NuoN) are shown in green. The distal glutamate residue (E407 on NuoM), the residue in question, is shown in red. Other titratable residues (D400^L^, E72^K^, and E36^K^) and residues of the E-channel are shown in black, bridging histidine residues in orange. Putative proton pathways are indicated with blue arrows. The central axis of charged and polar residues is indicated with a double arrow in orange. (*A*) The structure of the *E. coli* complex I was derived by in silico modeling with AlphaFold2 (63, 64). The subunits of the peripheral arm are shown in tan. Functional important residues involved in Q binding are shown in orange. The NADH and quinone binding sites, as well as the electron transfer pathway are indicated with red arrows. (*B*) Structure of the membrane arm of *E. coli* complex I (PDB ID code 3RKO). Putative proton pathways are indicated with broken arrows.

The mechanism of proton translocation by complex I is still under debate (19–22). The membrane arm contains four distinct, yet interconnected proton pathways that are linked to the Q binding and reduction site (the Q cavity) by an unusual central axis of polar and charged amino acid residues located approximately in the middle of the lipid bilayer (Fig. 1). This axis is oriented perpendicular to the membrane normal. The proton pathway closest to the Q cavity is called E-channel and is made up of subunits NuoH, NuoJ, and NuoK (23). The name stems from the presence of several conserved glutamate residues in these subunits that are most likely involved in proton transfer between the central hydrophilic axis and the Q cavity. Along the membrane arm, three further proton pathways line up provided by the antiporter-type subunits NuoN, NuoM, and NuoL (Fig. 1), which are arranged in an unusual face-to-back manner (23). The name derives from the homology of these subunits to those of multisubunit Na^+^/H^+^ antiporters. In complex I they are solely involved in proton translocation. These subunits are homologous to each other (24, 25), with each featuring two pseudosymmetrically arranged half-channels, of which the N terminus is open to the N-side and the C terminus to the P-side of the membrane (23). They comprise a conserved pattern of charged and polar residues that are thought to play an essential role in proton translocation along and across the membrane (23, 26–33). It is assumed that a defined charge distribution in the membrane arm is essential for proton translocation. Each of the antiporter subunits contains a conserved lysine/glutamate or lysine/aspartate ion pair, a conserved central lysine residue, and a distal lysine (NuoL and NuoN) or glutamate (NuoM) residue (Fig. 1). The distal lysine/glutamate residues are bridged to the central lysine residue by at least one conserved histidine residue (23). The central lysine residue is proposed to be protonated when the ion pair is closed (9, 26). The closed state describes a strong salt bridge between the residues of the ion pair. It is further assumed that quinone chemistry leads to a proton transfer to NuoK, causing the opening of the ion pair on NuoN (Fig. 1). The central lysine residue then transfers its proton to the distal residue, which then opens the ion pair at the next antiporter subunit (9). Finally, the proton is released from D400^L^ (the superscript refers to the name of the *E. coli* complex I subunit) to the P-side (Fig. 1). Proton release at D400^L^ triggers the reprotonation of the central lysine residues from the N-side in sequence, coupled with the proton release of the distal residues to the P-side (Fig. 1).

Remarkably, the distribution of the conserved titratable amino acid residues within NuoL, NuoM, and NuoN is asymmetric because NuoL and NuoN include a conserved distal lysine residue (K399^L^ and K395^N^), while NuoM, located between NuoL and NuoN, contains a conserved distal glutamate residue (E407^M^) (23). It was proposed that charge asymmetry within the membrane arm is needed to prevent electrostatic imbalance in the membrane arm and to achieve an asynchronous charge transfer within the antiporter subunits (31). Consequently, a variant with a symmetric charge distribution should not be able to catalyze proton translocation.

To test this hypothesis, we replaced the conserved E407^M^ residue by a lysine residue. The resulting E407K^M^ variant was stably assembled and showed a slightly diminished electron transfer activity. However, when reconstituted into liposomes, proton translocation was clearly diminished resulting in an H^+^/e^−^ stoichiometry of about 1.5, assuming a wild-type stoichiometry of 2 (33–35). Thus, the variant was capable of catalyzing redox driven proton translocation although less efficient. This finding is consistent with two models of proton translocation and renders the recently proposed “ND5-only” (31) model unlikely, as explained in the *Discussion*.

## Results

### Conservation of Amino Acid Residues.

Multiple sequence alignments showed the conservation of a glutamate residue at position 407 (*E. coli* numbering) within the NuoM homologs and the conservation of a lysine residue at positions 399 (NuoL) and 395 (NuoN) (*SI Appendix*, Fig. S1*A*) (23). Sequence comparison further showed that the conserved glutamate residue in NuoM is placed at the homologous position of the conserved lysine residue in NuoL and NuoM (Fig. 1 and *SI Appendix*, Fig. S1*B*) (23). It is assumed that each of these residues releases a proton to the P-side in the course of proton translocation (9, 21–23, 26–31). To answer the question of whether E407^M^ is essential for proton translocation, it was replaced by a lysine residue in the sequence of *E. coli* NuoM.

### Generation and Growth of the E407K^M^ Mutant.

The point mutation E407K^M^ was introduced into the pBAD*nuo*_His_ expression plasmid that encodes the entire *E. coli nuo*-operon under the control of the inducible P_BAD_ arabinose promoter (36). Possible recombination with the chromosomal wild-type allele during cloning was prevented by using strain DH5αΔ*nuo* as cloning host (36). The expression strain BW25113Δ*ndh nuo:npt*II_FRT was transformed with expression plasmids either encoding the parental genes or the ones with the mutation. The expression strain lacks the chromosomal gene of the alternative NADH dehydrogenase (*ndh*) (37) and the chromosomal *nuo*-operon encoding complex I is replaced by a resistance cartridge (*ntp*II) by λ-red–mediated recombination (38). Consequently, NADH-induced activities of membranes exclusively reflect activities of complex I encoded on the plasmid.

**Fig. 2.**
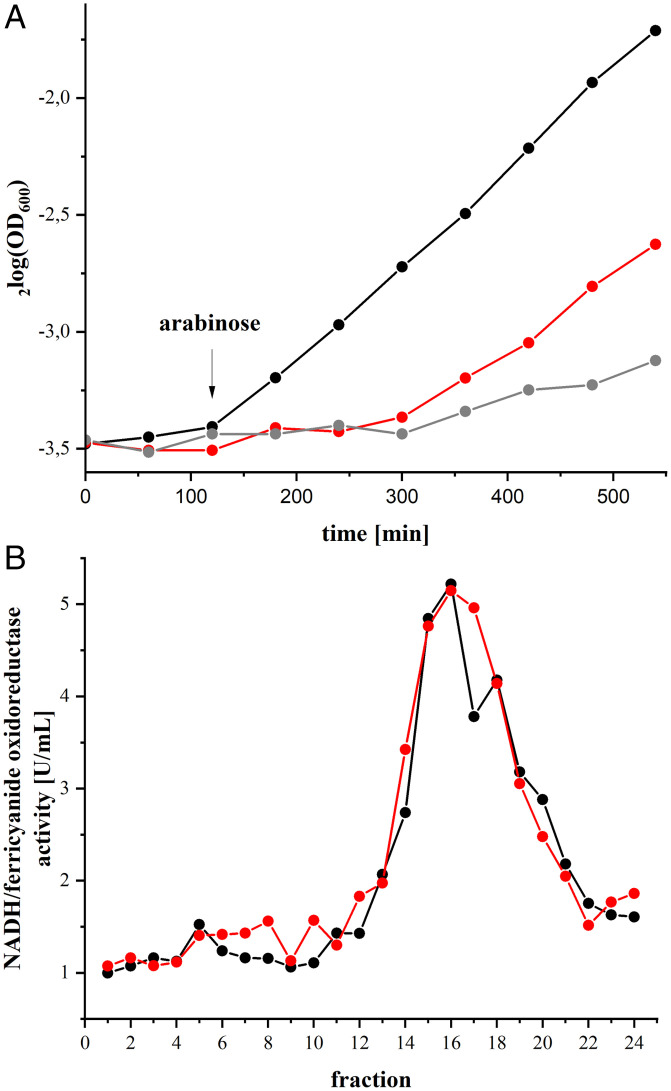
Growth of the mutant strain and stability of the E407K^M^ variant. (*A*) Growth of the host strain (gray) transformed either with the expression plasmid encoding the parental *nuo*-genes (black) or with the one encoding the E407K^M^ mutation (red) in minimal medium with acetate as sole carbon source. The black arrow indicates the induction of gene expression after 3 h growth by an addition of 0.02% l-arabinose. (*B*) Sucrose gradient of detergent solubilized membranes from wild-type (black) and the E407K^M^ (red) mutant strain. The NADH/ferricyanide oxidoreductase activity of each fraction is shown; the activities are normalized to 20-mg membrane protein extract applied on each gradient.

The host strain transformed either with the expression plasmid encoding the parental *nuo*-genes or with the one with the E407K^M^ mutation was grown in minimal medium with acetate as nonfermentable carbon source. A functionally intact complex I is required for fast growth to high OD values in this medium, because it maintains a low NADH/NAD^+^ ratio, which is essential to preserve the TCA cycle and contributes to the PMF (39, 40). The strain producing wild-type complex I grew the fastest, while the strain producing the E407K^M^ variant grew initially as slow as the host strain that was used as control. However, the mutant strain started to grow faster after ∼5 h but still significantly slower than the parental strain (Fig. 2). Thus, the E407K^M^ mutation affects complex I activity in the membrane.

### Assembly, Stability, and Activity of the Variant in Cytoplasmic Membranes.

Cells were disrupted and cytoplasmic membranes were obtained by differential centrifugation. The amount of the complex in the membranes was assessed from the artificial NADH/ferricyanide oxidoreductase activity. This activity is not coupled with Q reduction and proton translocation (41, 42) and it solely depends on the presence of the flavin cofactor, the amount of which is not affected by the mutation. Indeed, the NADH/ferricyanide oxidoreductase activity of the membranes from the mutant strain did not significantly differ from that of the parental strain (Table 1). The physiological activity was determined by measuring NADH oxidase activity. In this assay, NADH is oxidized by complex I in a rate-limiting step and the produced quinol (QH_2_) is reoxidized by the terminal quinol oxidases that utilize the electrons to reduce oxygen to water. The NADH oxidase activity of the membranes from the mutant strain was slightly reduced to ∼83% of that of the parental strain (Table 1). The activity curve of the NADH oxidase activity of both strains was linear, demonstrating that substrate binding was not affected by the mutation, as expected (*SI Appendix*, Fig. S2*A*). Thus, the E407K^M^ mutation did not influence the amount of complex I in the membrane and slightly diminished its electron transfer activity.

**Table 1.** NADH oxidase and NADH/ferricyanide oxidoreductase activity of cytoplasmic membranes from *E. coli* wild-type and mutant strain

	NADH oxidase activity		NADH/ferricyanide oxidoreductase activity	
	U/mg	%	U/mg	%
Wild-type	0.261 ± 0.020	100 ± 9	2.8 ± 0.3	100 ± 11
E407K^M^	0.213 ± 0.025	83 ± 12	2.7 ± 0.3	96 ± 11

The data were obtained from three measurements of two biological replicates.

The assembly of the complex in the mutant strain and its stability in the presence of the detergent LMNG (2,2-didecylpropane-1,3-bis-β-d-maltopyranoside) was investigated by sucrose gradient density centrifugation (Fig. 2*B*). Cytoplasmic membranes of cells grown in autoinduction-medium were incubated with LMNG and the cleared extract was loaded onto gradients from 5 to 30% (wt/vol) sucrose (43). Solubilized membrane proteins were separated from each other by centrifugation and the gradients were fractionated in 1-mL portions. The position of the complex and its variant in the gradient was determined by its NADH/ferricyanide oxidoreductase activity. The parental complex sedimented around fraction 16 (Fig. 2*B*). The variant protein sedimented at the same position of the gradient with approximately the same total activity as expected from the NADH/ferricyanide oxidoreductase activity of membranes (Table 1). Thus, the E407K^M^ variant was fully assembled in the membrane and stably extracted with LMNG.

### Preparation of Complex I and the E407K^M^ Variant.

Cells were grown in autoinduction-medium and cytoplasmic membranes were obtained by differential centrifugation (43). Membrane proteins were extracted with LMNG and the complex and the variant were prepared to homogeneity by affinity and size-exclusion chromatography (*SI Appendix*, Fig. S3 *A* and *B*) (17). From 50-g cells, 7- to 9-mg protein was obtained. The elution profiles of the complex and the variant showed no significant differences. The preparation of the parental complex and that of the variant contained all complex I subunits and the protein band pattern was virtually identical (*SI Appendix*, Fig. S3*C*).

### Catalytic Activities of the Preparations.

The preparations of the E407K^M^ variant had an insignificantly higher specific NADH/ferricyanide oxidoreductase compared to the complex (Table 2). The NADH:decyl-Q oxidoreductase activity was measured in the presence of quinol *bo*_3_ oxidase used as a quinone regenerating system, as described previously (17). The kinetic traces showed no lag-phase, indicating an unrestricted substrate binding and product release (*SI Appendix*, Fig. S2*B*). The E407K^M^ variant showed a specific activity of 80% of the activity of the parental strain (Table 2). This is in accordance with the NADH oxidase activity of the mutant membranes that was diminished to approximately the same extent (Table 1).

**Table 2.** NADH:decyl-Q and NADH/ferricyanide oxidoreductase activity of the preparations of complex I and the E407K^M^ variant

	NADH:Q oxidoreductase activity		NADH/ferricyanide oxidoreductase activity	
	U/mg	%	U/mg	%
Wild-type	32.8 ± 1.9	100 ± 6	107.0 ± 3.3	100 ± 3
E407K^M^	26.2 ± 2.3	80 ± 9	114.9 ± 4.7	107 ± 4

The data were obtained from three measurements of two biological replicates.

**Fig. 3.**
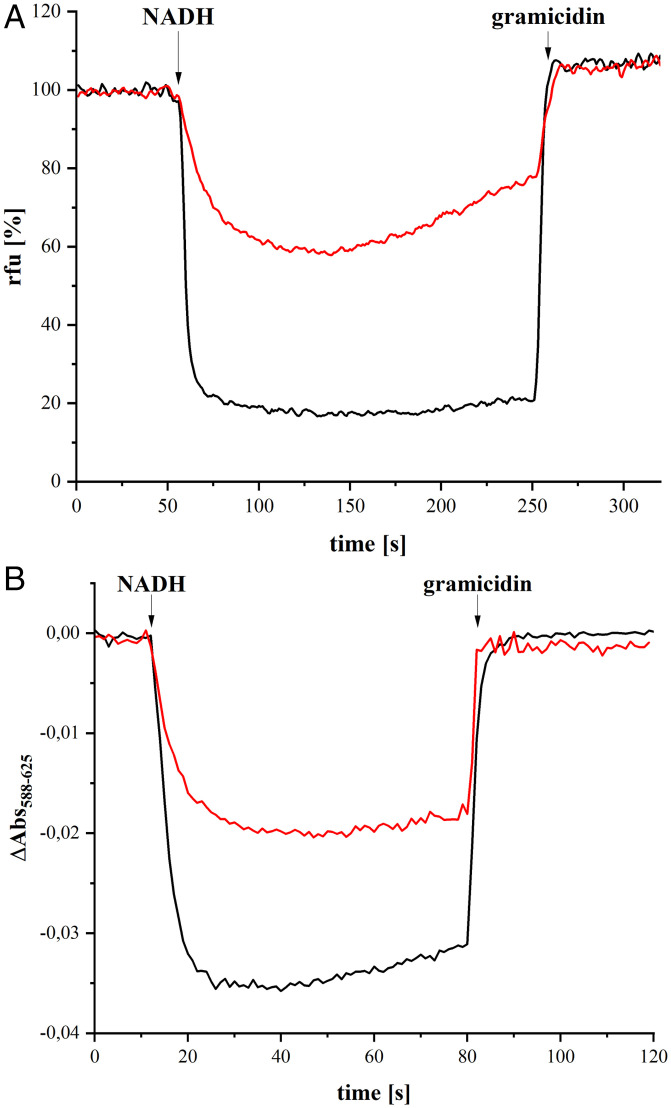
Generation of a PMF by *E. coli* complex I (black) and the E407K^M^ variant (red) reconstituted into liposomes. (*A*) Generation of a ΔpH measured as quench of the ACMA fluorescence. The reaction was started by addition of 130 µM NADH. Addition of gramicidin dissipated the proton gradient. (*B*) Generation of a ΔΨ measured as the decrease of the oxonol VI absorbance difference (588 nm to 625 nm). The membrane potential was dissipated by addition of gramicidin.

To measure proton translocation, complex I and the E407K^M^ variant were reconstituted into preformed liposomes, as described previously (44). The orientation of the enzymes in the liposomes was determined by measuring the NADH/ferricyanide oxidoreductase activity of intact proteoliposomes and an aliquot that was disintegrated by an addition of 2% detergent. The share of the accessible NADH binding sites was similar in the proteoliposome preparation of the complex and the variant (Table 3). The NADH:decyl-Q oxidoreductase activity of the preparation reconstituted into liposomes was determined by following the decrease of the NADH concentration at 340 nm. Due to the lack of a quinone regenerating system, the activities were lower and amounted to 0.19 (± 0.02) U/mg for the complex and to 0.16 (± 0.04) U/mg for the variant. Thus, the variant showed overall 84% of the electron transfer activity of complex I. Correcting this for the accessible NADH-binding sites in liposomes (Table 3) yields a value of 76%. These proteoliposome preparations were used in two different assays (Fig. 3).

**Table 3.** NADH-induced quench of ACMA fluorescence and decrease in oxonol absorbance due to the activity of complex I and the E407K^M^ variant reconstituted into liposomes

	ACMA quench		Decrease in oxonol absorbance		Accessible NADH sites
	%	% of WT	ΔA_588–625_	% of WT	%
Wild-type	80.0 ± 3	100 ± 4	0.0304 ± 0.001	100 ± 3	48 ± 5
E407K^M^	41.2 ± 4	52 ± 10	0.0163 ± 0.006	54 ± 3	53 ± 5

The data were obtained from three measurements of two biological replicates.

Proton pumping was measured by following fluorescence quenching of ACMA (9-amino-6-chloro-2-methoxyacridine). An addition of NADH led to an acidification of the proteoliposomes lumen due to proton pumping of the incorporated complex and the variant, respectively. However, the rate and the extent of fluorescence quenching of the proteoliposomes containing the E407K^M^ variant was reproducibly lower compared to those containing complex I. The signals obtained with both preparations were sensitive to an addition of gramicidin, demonstrating the presence of a proton gradient. The variant showed 52% of the ACMA quench of the complex (Table 3). An addition of valinomycin accelerated the reaction, but did not alter the relative quench ratio observed with the complex and the variant (*SI Appendix*, Fig. S4).

The membrane potential was directly determined by following the decrease in the absorbance of the potential sensitive dye oxonol VI (Fig. 3). As with the ACMA measurements, the signal’s intensity and the rate of formation was approximately halved with the proteoliposomes containing the E407K^M^ variant compared to the signal obtained with proteoliposomes containing complex I. Again, the signals obtained with both preparations were fully sensitive to an addition of gramicidin that abolishes the membrane potential. The oxonol absorbance of proteoliposomes containing the variant was decreased to 54% compared to those containing complex I (Table 3).

The data show that the E407K^M^ variant is capable of catalyzing redox-driven proton-translocation. In proteoliposomes, the electron transfer activity of the variant amounts to 84% of that of the complex. However, the variant liposomes contain a larger share of active enzyme due to its orientation within the membrane and the membrane impermeability of NADH. Taking the different accessibility of the NADH binding sites into account (Table 3), the variant exhibits 76% of the electron transfer activity of the complex in liposomes. Correcting the values of proton translocation activity (Table 3) with the electron transfer activity, the ACMA fluorescence quench of the variant reaches 68% (SD: ± 10.1%) of that of complex I and the oxonol absorbance amounts to 71% (SD: ± 14.8%). As measurements with proteoliposomes tend to underestimate the magnitude of proton translocation due to an inherent leakiness (33), the E407K^M^ variant presumably translocates 75% of the number of protons per electrons compared to complex I. Assuming a stoichiometry of 2 for the latter (33–35), the H^+^/e^−^ stoichiometry of the E407K^M^ variant is diminished to 1.5.

### Effect on p*K*a Values.

**Fig. 4.**
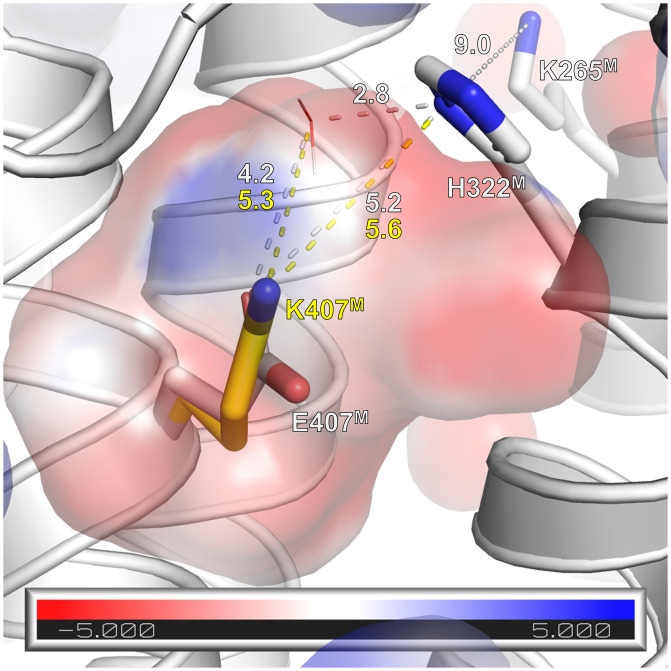
Environment of position 407^M^ in *E. coli* complex I (from PDB ID code 3RKO). The position of K265^M^, H322^M^, and E407^M^ and K407^M^ are shown. K265^M^ most likely transfers a proton via H322^M^ to position 407^M^. Potential surface is shown around the mutated position with red indicating a negative charge and blue a positive charge. The position of a structurally resolved water molecule is included and distances are provided in ångstroms. Proton transfer from K265^M^ might be enabled by further water molecules that are not resolved in the structure (22, 30).

The altered H^+^/e^−^ stoichiometry of the variant may be due to a change in the local p*K*a value at position 407^M^ caused by the substitution of the glutamate by a lysine residue. We calculated the p*K*a value at this position in comparison to that of K265^M^, the proposed proton donor to E407^M^ using the program PROPKA 3.0 (45, 46). The mutation E407K^M^ was introduced in silico into the structure of the *E. coli* membrane arm (Protein Data Bank [PDB] ID code 3RKO) (23) followed by energy minimization and H-bond optimization. The p*K*a of K265^M^ was determined to 6.8 in the original and the variant structure. The p*K*a value of the original E407^M^ was calculated to be 7.6, which raises the question of whether the membrane arm actually exhibits charge asymmetry, as suggested previously (31), since the lysine residues at the homologous position in NuoN is very similar (K395; p*K*a = 7.4), while that in NuoL (K399; p*K*a = 6.2) is more acidic. The latter is most likely due to its close vicinity to D400^L^, the proposed proton exit. Beyond that, the p*K*a of the substituted K407^M^ exhibits a p*K*a of 6.8. Consequently, the mutation shifted the p*K*a at position 407^M^ by nearly one pH unit, which renders proton transfer from K265^M^ to position 407^M^ more unlikely in the variant. This shift is presumably caused by the overall negative electrostatic surface potential in the environment of the carboxylic group of E407^M^ (Fig. 4). Due to charge repulsion, the protonation of the carboxylate group of E407^M^ is promoted. Modeling the lysine residue into the available space brings the ε-amino group close to a small positively charged surface patch favoring its neutral state (Fig. 4). Proton transfer from K265^M^ to position 407^M^ might be facilitated by H322^M^ (Fig. 4) and water molecules, one of which is found in the structure (22, 23). Remarkably, the E407K^M^ mutation also had a strong impact on H322^M^ and neighboring residues, lowering the p*K*a by approximately two pH units. This could contribute to a significantly disturbed proton transfer from K265^M^ to K407^M^ and, furthermore, that K407^M^ is less suitable for storing protons than E407^M^.

## Discussion

Although several structures of complex I from various species are available at resolutions ranging from 2.3 to 4.5 Å, the molecular mechanism of proton translocation is still not understood. Proposals have been made to distinguish the deactive from the active state of the complex (31, 47, 48), the binding sites of several Q-site inhibitors have been determined (49–51), and the structure of the complex was obtained in the presence of NADH, quinone, both substrates, and under turnover conditions (31, 52, 53). Several models for proton translocation were proposed mainly based on the combination of structural data and molecular dynamics simulations (9, 20–22, 30–32, 54). However, one has to keep in mind that proton translocation might be accomplished via several short-lived conformational states of higher energy, which might not be detectable by cryoelectron microscopy (cryo-EM). We can also not exclude that the presence of the biological membrane and the PMF are needed to detect such intermediate states. A significant complementary method to confirm or disprove proposals on the enzyme’s mechanism is therefore the kinetic investigation of mutants, in which key amino acids have been exchanged.

**Fig. 5.**
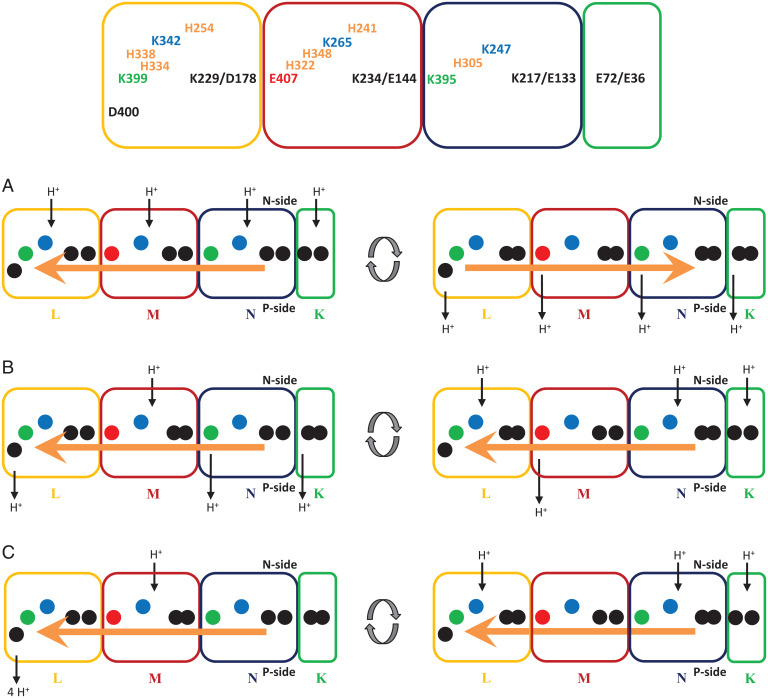
Putative proton pumping mechanisms of complex I. Subunits NuoL (yellow), NuoM (red), NuoN (blue), and NuoK (green) are shown as blocks, the titratable amino acid residues as dots. The name of the individual residues are given in the *Top* scheme. The ion pairs are shown in black, the opened and closed conformations are indicated by separated and fused dots, respectively. The central lysine residues are shown in blue, the distal lysine residues (NuoL and NuoN) in green and the distal glutamate residue (NuoM) in red. Putative proton pathways are indicated by black arrows, forward (or first) and reverse (or second) power strokes are indicated by orange arrows. (*A*) Two-stroke mechanism with the propagation of a forward and a reverse electrostatic wave, leading to proton uptake and release in all three antiporter subunits. (*B*) Asynchronous proton uptake and release by two strokes moving in the same direction. (*C*) Same as in *B*, but all protons are released to the P-side by NuoL (ND5-only model). See *Discussion* for details.

Position E407^M^ is such a key amino acid because the conserved glutamate residue is thought to introduce charge asymmetry into the membrane arm of complex I as the common charged distal residue is a lysine in NuoL and NuoN, while it is a glutamate residue in NuoM (Fig. 5) (23, 31). To the best of our knowledge, this residue has not been studied by mutational analysis so far. The E407K^M^ mutation should introduce charge symmetry into the membrane arm and should therefore influence proton translocation.

The most recent proposal for proton translocation by complex I is a two-stroke mechanism, with one stroke initiated by the reduction and protonation of Q, leading to proton release from the central hydrophilic axis to the P-side, and a second stroke due to the movement of the QH_2_ in the cavity, leading to a proton uptake to the central axis from the N-side (31). Both strokes lead to conformational changes that are first transferred to the E-channel and finally lead to a proton transfer within the ion pairs of the antiporter subunits. Protons are proposed to be translocated in a uniform manner across all antiporter subunits by pure electrostatic interactions (31). Due to the change of charges within the K217^N^/E133^N^ ion pair, the charge of the aforementioned conserved amino acids and the bridging histidine residues changes accordingly (Fig. 5). This kind of change continues in the same manner throughout the entire membrane arm. The uneven charge distribution within the antiporter subunits is supposed to prevent an electrostatic imbalance in the membrane arm provided that NuoM operates asynchronously to NuoL and NuoN. The presence of the distal glutamate residue in NuoM was proposed to be causative for the “out-of-sync” operation of the antiporter subunits. According to this model, one proton per antiporter subunit is translocated across the membrane. Hampering proton transfer from NuoM to NuoL by the E407K^M^ mutation would accordingly disconnect NuoL from changes in the charge of the conserved amino acid residues propagating along the membrane arm. Proton release triggered by the first stroke and proton uptake by the second stroke would no longer be transmitted to NuoL, thus diminishing the H^+^/e^−^ stoichiometry from 2 (complex I) (33–35) to 1.5 (E407K^M^). This diminished stoichiometry of 1.5 H^+^/e^−^ is thus in agreement with the measured stoichiometry reported here, although no asynchronous operation of NuoM, a central feature of the proposed mechanism, is needed as the E407K^M^ variant with a symmetric charge distribution within the antiporter subunits is obviously capable of proton pumping.

The same authors also proposed a “ND5-only mechanism” (31). Subunit ND5 is the homolog of NuoL in mitochondrial complex I (Fig. 5). Here, the two-stroke mechanism triggering proton translocation, as described above, applies; but according to this mechanism, all protons are translocated across the membrane only by the most distal subunit NuoL (ND5). This proposal is based on the observation that the E-channel as well as NuoM and NuoN lack proton pathways to the P-side in cryo-EM structures. On the contrary, the distal conserved lysine residue, K399^L^ (K392^ND5^), was found to be well connected to the P-side by conserved D400^L^ (D393^ND5^) and by several water molecules (Fig. 5). Furthermore, no conformational changes of the antiporter subunits were observed when the sample was frozen for EM analysis in the presence of NADH and decyl-Q. It was concluded that the accessibility of protons from NuoM and NuoN toward the P-side is not altered during turnover. As protons may travel all along the central hydrophilic axis of the membrane arm and because NuoM and NuoN may not have contact to the P-side, it was proposed that all protons are pumped to the P-side by NuoL. Accordingly, four protons are taken up from the N-side, most likely by NuoL and NuoM, and are further distributed along the central hydrophilic axis to the distal NuoL, probably including a temporal storage on the conserved bridging histidine residues (31).

This proposal is in contrast to findings obtained with deletion mutants in *Yarrowia lipolytica* and *E. coli* complex I (55, 56). In *E. coli*, the deletion of *nuoL* led to the assembly of stable complex just lacking NuoL that was capable to pump protons with a H^+^/e^−^ stoichiometry of about 1 (55). Similar results were obtained with *Y. lipolytica*: deletion of a gene encoding a small accessory subunit led to the formation of a complex that lacked NuoL and NuoM (ND5 and ND4). This complex I variant also showed a reduced stoichiometry of 1 (56). However, it can be argued that deletion of one or two of the antiporter subunits may distort the well-balanced architecture of the membrane arm, leading to the presence of artificial proton pathways that are not existent in the holo-complex.

In contrast, it is highly unlikely that the E407K^M^ point mutation described here will lead to significant structural rearrangements. The calculated p*K*a shift at positions 407^M^ and H322^M^ induced by the mutation might interrupt the postulated free distribution of protons along the central hydrophilic axis (31). If all protons were released to the P-side by NuoL, the mutation would lead to a complete loss of proton translocation. If, instead, the shift in p*K*a was not sufficient to interrupt the free distribution of protons along the central axis, the mutation would have no effect on the H^+^/e^−^ stoichiometry according to the ND5-only mechanism. Remarkably, the mutation diminished the H^+^/e^−^ stoichiometry, which is inconsistent with the predicted effects. Thus, our results obtained with the E407K^M^ variant argue strongly against the proposed ND5-only mechanism.

Based mainly on computational work, an initial proposal suggests a one-stroke mechanism with each of the antiporter subunits and the E-channel pumping one proton per stroke (9). It assumes a progression of an “electrostatic wave” triggered by the movement of a QH_2_-species in the Q cavity that leads to the opening of the K217/E133^N^ ion pair (Fig. 5). The protonated central K247^N^ transfers its proton to the distal K395^N^, which leads to an opening of the lysine/glutamate ion pair in NuoM (E144^M^/K234^M^). Accordingly, a “wave” of changes in the electrostatic properties of the titratable amino acid residues propagates across the entire membrane arm. The distal K399^L^ will pass on its proton to conserved D400^L^ that releases this proton to the P-side. This de-protonation induces a “back wave” leading to a protonation of the central lysine residues from the N-side, closing of the lysine/glutamate ion pairs and a proton release from E407^M^, K395^N^, and probably from E72^K^ or E36^K^ to the P-side. This proposed mechanism involves the opening and closing of the conserved ion pairs in the antiporter subunits and a concerted opening and closing of the proton pathways leading, on the one hand, from the N-side to the central hydrophilic axis of the membrane arm, and on the other hand, from there to the P-side. As these structural changes have not been observed by cryo-EM, this mechanism was considered unlikely (31). However, it is also possible that these conformational changes represent short-lived conformational states that are thermodynamically unfavorable and might not be detectable by cryo-EM.

According to this mechanism, the de-protonation of the central K265^M^ will close the proton pathway in NuoM to the N-side and the protonation of E407^M^ causes an opening of the K229^L^/D178^L^ ion pair. In the E407K^M^ variant, K407^M^ might not receive a proton from K265^M^ due to the change of the p*K*a value at this position and of H322^M^. Consequently, the proton on K265^M^ might equilibrate via the open half-channel with the N-side, initiating the back wave and leaving the K229^L^/D178^L^ ion pair closed. Such a scenario should result in a H^+^/e^−^ stoichiometry of 1. However, it appears possible as well that K265^M^ transfers the proton to K407^M^, but then, due to the lowered p*K*a, K407^H+M^ would favor proton translocation to the P-side over proton storage. Here, the K229^L^/D178^L^ ion pair would remain closed. This would lead to a H^+^/e^−^ stoichiometry of 1.5. Indeed, the measured H^+^/e^−^ stoichiometry of 1.5 is in good agreement with the proposed mechanism provided that K407^M^ accepts a proton from K265^M^. An alternative explanation is that K407^H+M^ is no longer capable to donate its proton to the P-side and donates its proton to K265^M^ during the “back wave reaction.” In total, the stoichiometry would be diminished to 1.5 as well.

## Materials and Methods

### Strains, Plasmids, and Oligonucleotides.

A derivative of *E. coli* strain BW25113 (35) chromosomally lacking the gene *ndh* was used as host to overproduce complex I (17). In this strain, the chromosomal *nuo* operon was also replaced by a resistance cartridge (*npt*II). *E. coli* strain DH5αΔ*nuo* was used for site-directed mutagenesis (33). Oligonucleotides were obtained from Sigma-Aldrich (*SI Appendix*, Table S1). Restriction enzymes were obtained from Fermentas.

Plasmid pBAD*nuo_His_* (57) was used to introduce the respective point mutation on *nuoM* by site-directed mutagenesis according to the QuikChange protocol (Stratagene). A silent mutation was introduced generating a new restriction site close to the point mutation to identify positive clones by restriction analysis. Primer pair nuoM_E407K (*SI Appendix*, Table S1) was used to generate the plasmid pBAD*nuo _His_nuoF* E407K^M^. The PCR was performed using the KOD Hot Start DNA Polymerase (Novagen). Mutations were confirmed by DNA sequencing (GATC Eurofins).

### Cell Growth and Preparation of Cytoplasmic Membranes.

Strains were grown aerobically at 37 °C while agitating at 180 rpm. Cells were grown in baffled flasks containing minimal medium with 25 mM acetate as sole carbon source (58). Expression of the *nuo* operon was induced after 3 h of growth by an addition of 0.02% (wt/vol) l-arabinose. For protein preparation, cells were grown in a rich autoinduction medium (1% [wt/vol] peptone, 0.5% [wt/vol] yeast extract, 0.4% glycerol, 25 mM Na_2_HPO_4_·2 H_2_O, 25 mM KH_2_PO_4_, 50 mM NH_4_Cl, 5 mM Na_2_SO_4_, 2 mM MgSO_4_·7 H_2_O, 0.2% [wt/vol] l-arabinose, 0.05% [wt/vol] glucose, 30 mg/L Fe-NH_4_-citrate, 0.5 mM l-cysteine, 50 mg·L^−1^ riboflavin) containing chloramphenicol (34 µg/mL) (59). Cells were harvested by centrifugation (5,700 × *g*, 15 min, 4 °C; Avanti J-26 XP, Beckman Coulter; Rotor JLA 8.1000) in the exponential phase yielding between 6 and 7 g cells/L. All further steps were carried out at 4 °C. Sedimented cells were suspended in a fivefold volume of buffer A (50 mM MES/NaOH, 50 mM NaCl, pH 6.0) containing 0.1 mM PMSF and a few grains of DNaseI. They were disrupted by three passages through an HPL-6 (Maximator, 1,000 to 1,500 bar). Cell debris was removed by centrifugation (12,074 × *g*, 20 min, 4 °C; Rotor JA25.50, Beckman; Avanti J-26S XP, Beckman Coulter). Cytoplasmic membranes were obtained from the supernatant by ultracentrifugation (160,000 × *g*, 70 min, 4 °C; L8-M Ultrafuge, Beckman; Rotor 60 Ti). Sedimented membranes were suspended in an equal volume (1:1 [wt/vol]) of buffer A* (buffer A with 5 mM MgCl_2_) containing 0.1 mM PMSF.

### Electron Transfer Assays.

Activity assays were performed at 30 °C. The NADH oxidase activity of cytoplasmic membranes was determined by a Clarke-type oxygen electrode (DW1, Hansatech) monitoring the decrease in oxygen concentration in the buffer. The electrode was calibrated by an addition of a few grains sodium dithionite to air saturated buffer. The difference in current before and after reduction was attributed to 237 mM oxygen (60). The assay contained 5-µL cytoplasmic membranes (80 to 90 mg/mL) in 2 mL buffer A*. After equilibration, the reaction was started by an addition of 1.25 mM NADH. The NADH/ferricyanide oxidoreductase activity was determined as decrease in the absorbance of ferricyanide at 410 nm with a diode-array spectrometer (QS cuvette, *d* = 1 cm, Hellma; TIDAS II, J&M Aalen) using an ε of 1 mM^−1^cm^−1^ (61). The assay contained 2-µL membrane suspension or 0.1 µL complex I and 1 mM K_3_[Fe(CN)_6_] in buffer A*. The reaction was started by an addition of NADH (0.2 mM, final concentration). The NADH:decyl-Q oxidoreductase activity was measured as decrease of the NADH concentration at 340 nm using an ε of 6.3 mM^−1^cm^−1^ (QS cuvette, *d* = 1 cm, Hellma; TIDAS II, J&M Aalen). The assay contained 60 µM decyl-Q, 2 µg complex I and a tenfold molar excess (5 µg) *E. coli* cytochrome *bo_3_* oxidase in buffer A*_MNG_ (buffer A* with 10% (vol/vol) glycerol and 0.005% (wt/vol) LMNG (Anatrace). The reaction was started by an addition of 150 µM NADH (62).

### Sucrose Gradient Centrifugation.

Membrane proteins were extracted by an addition of 1% (wt/vol) LMNG to a membrane suspension (80 to 90 mg/mL) in buffer A* (17). After incubation for 1 h at 4 °C, the suspension was centrifuged for 20 min at 160,000 × *g* and 4 °C (Rotor 60Ti, L8-M Ultrafuge, Beckman). Next, 1 mL of the supernatant was loaded onto 24-mL gradients of 5 to 30% (wt/vol) sucrose in A*_LMNG_ (buffer A* with 0.05% [wt/vol] LMNG) and centrifuged for 16 h at 140,000 × *g* (4 °C, Rotor SW28, L8-M Ultrafuge, Beckman). The gradients were fractionated into 1-mL portions and the NADH/ferricyanide oxidoreductase activity of each fraction was determined.

### Preparation of Complex I and the Variant.

All steps were carried out at 4 °C as described previously (17): 2% (wt/vol) LMNG (final concentration) was added to the membrane suspension (∼70 mg/mL) in buffer A*_pH6.8_. After 1-h incubation at room temperature with gentle stirring, the suspension was centrifuged for 20 min at 160,000 × *g* and 4 °C (Rotor 60Ti, L8-M Ultrafuge, Beckman). The supernatant was filtered (Filtropur S0.45; Sarstedt), diluted to 150 mL, adjusted to 20 mM imidazole, and applied to a 35-mL ProBond Ni^2+^-IDA column (Invitrogen) equilibrated in binding buffer (A*_MNG_ with 20 mM imidazole, pH 6.8). The column was washed with binding buffer and with binding buffer containing 116 mM imidazole until the absorbance at 280 nm dropped below 500 mAu. Bound proteins were eluted with binding buffer containing 308 mM imidazole. Fractions containing NADH/ferricyanide oxidoreductase activity were pooled and concentrated by ultrafiltration in 100 kDa MWCO Amicon Ultra-15 centrifugal filter (Millipore) (43). The concentrate was applied onto a Superose 6 size-exclusion chromatography column (300 mL; GE Healthcare) equilibrated in buffer A*_MNG_. The fractions with highest NADH/ferricyanide oxidoreductase activity were pooled and concentrated as described above. The protein was either directly used or stored at −80 °C.

### Preparation of Liposomes and Measurement of Gradients.

*E. coli* polar lipids (25 mg/mL in CHCl_3_; Avanti) were evaporated and dissolved in five times the volume of lipid buffer (5 mM MES/NaOH, 50 mM NaCl, pH 6.7). The suspension was frozen in liquid nitrogen and thawed at 29 °C seven times to get unilamellar vesicles (44). The liposomes were extruded by at least 21 passes through an extruder (0.1 µM polycarbonate membrane; Mini Extruder, Avanti). For reconstitution, ∼0.5-mg complex I was mixed with reconstitution buffer (1:3 [vol/vol]) (10 mM Bis-Tris-propane/MES, 100 mM KCl, 73 mM sucrose, 2.5 mM MgSO_4_, 0.05% [wt/vol] l-α-phosphatidylcholine, 1.1% [wt/vol] *n*-octyl glucoside, 0.6% [wt/vol] sodium deoxycholate, 0.6% [wt/vol] sodium cholate, pH 7.5) and incubated for 5 min on ice. Next, 250-µL liposomes were mixed with 8 µL sodium cholate (20% [wt/vol]) and the liposomes and complex I in reconstitution buffer were mixed and incubated for 20 min at room temperature with occasional flicking of the tube. Liposomes were formed using a size-exclusion column (PD-10 Desalting Column, 8.3 mL, Sephadex G-25, GE Healthcare) equilibrated in lipid buffer to remove excess detergent. The eluate (1.2 mL) was centrifuged (4 °C, 200,000 × *g*, 30 min; Rotor, A-100, Airfuge, Beckman) and sedimented proteoliposomes were gently resuspended in 500-µL lipid buffer. They were used immediately in subsequent assays at 30 °C.

The generation of ΔpH was determined by monitoring the fluorescence quenching of the pH-sensitive dye ACMA (Sigma). The assay contained 100 µM decyl-Q (Sigma), 0.2 µM ACMA, and 50 µL proteoliposomes in ACMA buffer (10 mM Bis-Tris-propane/MES, pH 6.75, 100 mM KCl, and 2 mM MgCl_2_). The reaction was started by an addition of 130 µM NADH. Fluorescence was detected with a LS 55 Fluorescence Spectrometer (Perkin-Elmer) using excitation and emission wavelengths of 430 nm and 480 nm, respectively. An addition of 1-µg gramicidin (Sigma) was used to dissipate the ΔpH.

The generation of *Δ*Ψ was determined by monitoring the changes in absorption of the potential-sensitive dye oxonol VI (Sigma). The assay contained 0.5 µM oxonol VI, 50 µM Q_0_ and proteoliposomes in oxonol buffer (10 mM MES/KOH, pH 6.75, 2 mM MgSO_4_, 100 mM KCl, 10 mM NH_4_Cl). The reaction was started by an addition of 100 µM NADH. The absorbance difference at 588 minus 625 nm was measured with a diode-array spectrometer (QS cuvette, *d* = 1 cm, Hellma; TIDAS II, J&M Aalen). An addition of 1-µg gramicidin was used to dissipate *Δ*Ψ.

### In Silico Modeling of *E. coli* Complex I.

A structural model of the holo-complex I from *E. coli* was generated using AlphaFold 2.1.1 (63) in multimer mode (64). The complex was modeled in two separate segments, one comprising the membrane arm and the basal peripheral stem, with a total of 10 separate chains [i.e., NuoAB(CD)HIJKLMN]. In a separate run, we modeled the peripheral arm in an assembly of six subunits, NuoB(CD)EFGI. The full complex was then assembled with the overlapping subunits as a guide, avoiding the inclusion of distortions of subunits that were created by the absence of adjacent protomers. Following the assembly of the modeled protein subunits, the iron–sulfur clusters and FMN cofactor of the peripheral arm were modeled by hand and the entire complex was geometry-optimized using the Maestro Suite (Schrödinger).

### Other Analytical Methods.

Protein concentration was determined according to the biuret method using BSA as a standard (65). The concentration of purified complex I was determined by the difference of absorbance at 280 to 310 nm (TIDAS II, J&M Aalen) using an ε of 781 mM^−1^cm^−1^ as derived from the amino acid sequence (66). SDS/PAGE was performed according to Schägger and von Jagow (67) with a 10% separating gel and a 3.9% stacking gel. Multiple sequence alignments were performed using ClustalX 2.1 (68). Default values were used for the penalties for opening and extending gaps.

## Supplementary Material

Supplementary File

## Data Availability

All study data are included in the main text and/or *SI Appendix*. Genetic matrial can be made available upon reasonable request.
