# Supplementary material for: Respiratory complex I with charge symmetry in the membrane arm pumps protons
Source: Proc Natl Acad Sci U S A. 2022 Jun 27;119(27):e2123090119. doi: 10.1073/pnas.2123090119 (PMC9271201; doi:10.1073/pnas.2123090119)
Supplement: Supplementary File [file pnas.2123090119.sapp.pdf]

PNAS
www.pnas.org

Supplementary Information for
Respiratory complex I with charge symmetry in the membrane arm pumps protons

Franziska Hoeser, Hannes Tausend, Sinja Götz, Daniel Wohlwend, Oliver Einsle, Stefan Günther and Thorsten Friedrich

Email: Friedrich@bio.chemie.uni-freiburg.de

This PDF file includes:

Figures S1 to S4
Table S1

A)

NuoL/ND5/Nqo12

```
E. coli         HHEQNIFKMGGLRKSIPLVYLCFLVGGAALSALPLVTAGFFSKDEILAGAM-A--NGHINLMVAGLVGAFMTSLYTFRM 432
H. sapiens      NNEQDIRKMGGLLKTMPLTSTSLTIGSLALAGMPFL-TGFYSKDHIIETANMSYTNA--WALSITLIATSLTSAYSTRM 426
T. thermophilus GGEQDVRKMGGLWKHLPQTRWHALIGALALGGLPLL-SGFWSKDAILAATL-TYPFGGVGFYVGALLVAVLTAMYAMRW 420
Y. lipolytica   NESQDIRTYGGLLSYLPYTYICITIASLSLMAMPGL-TGYYTKDIIIESTYGSYSISNYVVYWIAYLSAVLTCVYSMKI 432
M. musculus     ADEQDIRKMGNITKIMPFTSSCLVIGSLALTGMPFL-TGFYSKDLIIEAINTCNTNA--WALLITLIATSMTAMYSMRI 426
B. taurus       NDEQDIRKMGGLFKAMPFTTTALIVGSLALTGMPFL-TGFYSKDLIIEAANTSYTNA--WALLMTLIATSFTAIYSTRI 426
                  .*:: . *.: . :* .     :.. :* .:* : :*:::** *:          .         : : :*. *: :
```

NuoM/ND4/Nqo13

```
E. coli         MGGLWSKMKWLPALSLFFAVATLGMPGTGNFVGEFMILFGSFQVVPVITVISTFGLVFASVYSLAMLHRAYFGKAKSQI 452
H. sapiens      SQGLQTLLPLMAFWWLLASLANLALPPTINLLGELSVLVTTFSWSNITLLLTGLNMLVTALYSLYMFTTTQWGSLTHHI 423
T. thermophilus YRGLAQSAPGLAALALILFLAMVGLPGLSGFPGEFLTLLGAYKASPWLAALAFLSVIASAAYALTAFQKTFWEEGGSGV 422
Y. lipolytica   YKGLTTYMPQLATYIIILSFANIGTPLTGNFTGEFLSLQGGFIRNPIIGGISCISVLLAAIYQLKLTNKLTGGISSIYM 440
M. musculus     ARGLQMVFPLMATWWLMASLANLALPPSINLMGELFITMSLFSWSNFTIILMGINIIITGMYSMYMIITTQRGKLTNHM 423
B. taurus       ARGLQTLLPLMATWWLLASLTNLALPPTINLIGELFVVMSTFSWSNITIILMGVNMVITALYSLYMLIMTQRGKYTYHI 423
                  **      :    ::  .: :. *    .: **:       :         :  ..:: :. * :           :
```

NuoN/ND2/Nqo14

```
E. coli         DADSLFSYRGLFWHRPILAAVMTVMMLSLAGIPMTLGFIGKFYVLAVGVQAHLWWLVGAVVVGSAIGLYYYLRVAVSLY 433
H. sapiens      SSTTTLLLSRTWNKLTWLTPLIPSTLLSLGGLPPLTGFLPKWAIIEEFTKNNSLIIPTIMATITLLNLYFYLRLIYSTS 301
T. thermophilus DRVPLEALRGLYRKDPLLGLAFLVAMLSLLGLPPLAGFWGKYLAFAEAARAGAWGVLVLALVTSAVSAYYYLGLGLAVF 382
Y. lipolytica   PIIYVNQLKGLIHDNAYLVLSMAIVVFSFIGIPPLLGFFGKLNILMSILNNGYYFISIVLIVASLISALYYLYLLNVSI 421
M. musculus     NSMTINSISLLWNKTPAMLTMISLMLLSLGGLPPLTGFLPKWIIITELMKNNCLIMATLMAMMALLNLFFYTRLIYSTS 301
B. taurus       STTTTLSLSHTWNKTPIMTVLILATLLSMGGLPPLSGFMPKWMIIQEMTKNNSIILPTFMAITALLNLYFYMRLTYSTT 301
                          .  :   :   ::*: *:*   **  *   :
```

B)

E. coli

```
NuoL  LRKSIPLVYLCFLVGGAALSALPLVTAGFFSKDEILAGAMANGH----INLMVAGLVGAF 423
NuoM  LWSKMKWLPALSLFFAVATLGMP-GTGNFVGEFMILFGSFQVV-----PVITVISTFGLV 430
NuoN  FWHR-PILAAVMTVMMLSLAGIP-MTLGFIGKFYVLAVGVQAHLWWLVGAVVVGSAIGLY 423
      :        :       .    :   .:*  *  .*..:   :*   ..          :  *  .  .*
```

H. sapiens

```
ND5   MPLT-----STSLTIGSLALAGMPFLTGFYSKDHIIETANMSYTNAWA-LS-ITLIATSL 418
ND4   LPLMAFWWLLASL-----ANLALPPTINLLGELSVLVTTF-SWSNITLLLTGLNMLVTAL 405
ND2   ----KLTWLTPLIPSTLLSLGGLPPLTGFLPKWAII-EEF-TKNNS-LIIP-TIMATITL 287
                     :     :  .:*    .:   :  ::      : .*     :     : . :*
```

T. thermophilus

```
Nqo12 GLWKHLPQTRWHALIGALALGGLPLLSGFWSKDAILAATLTYPFGGVGFYVGALLVAVLT 413
Nqo13 GLAQSAPGLAALALILFLAMVGLPGLSGFPGEFLTLLGAYK--ASPWLAA-LAFLSVIAS 405
Nqo14 GLYRKDPLLGLAFLVAMLSLLGLPPLAGFWGKYLAFAEAAR--AGAWGVLVLALVTSAVS 373
      ** :  *       *:  *:: *** *:** .:   :  :      .        *::    :
```

Fig. S1. Multiple sequence alignments A) of homologues of NuoL, M and N and B) between NuoL/NuoM/NuoN. The sequences from *E. coli, Homo sapiens, T. thermophilus, Y. lipolytica*, *Mus musculus and Bos taurus* are shown exemplarily. 85 sequences were used to generate the alignments. The positions of the conserved distal lysine and glutamate residues are marked in blue. The '*' indicates conserved positions, the ':' conservative substitutions.

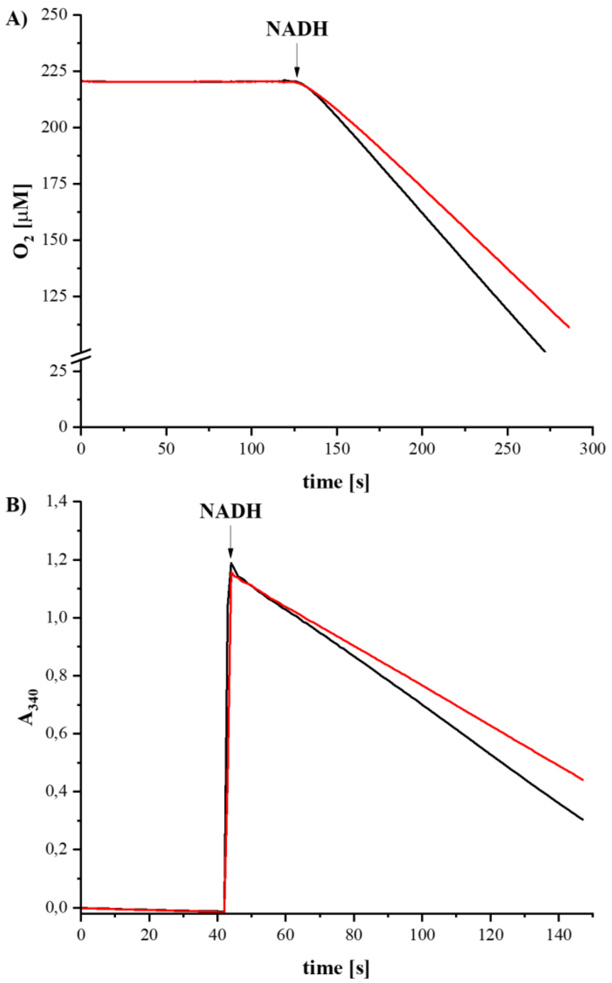

Fig. S2. NADH oxidase A) and NADH:Q oxidoreductase B) activity of complex I (black) and the E407K^{M} variant (red). The initial traces show no lag phase in both activities indicating an unrestricted access of the substrates to the enzyme and an unrestricted release of the products from the enzyme. In A), the decrease of the oxygen concentration is measured with a Clark-type electrode and the activity is started by addition of NADH. In B), the decrease of the NADH concentration is measured at 340 nm.

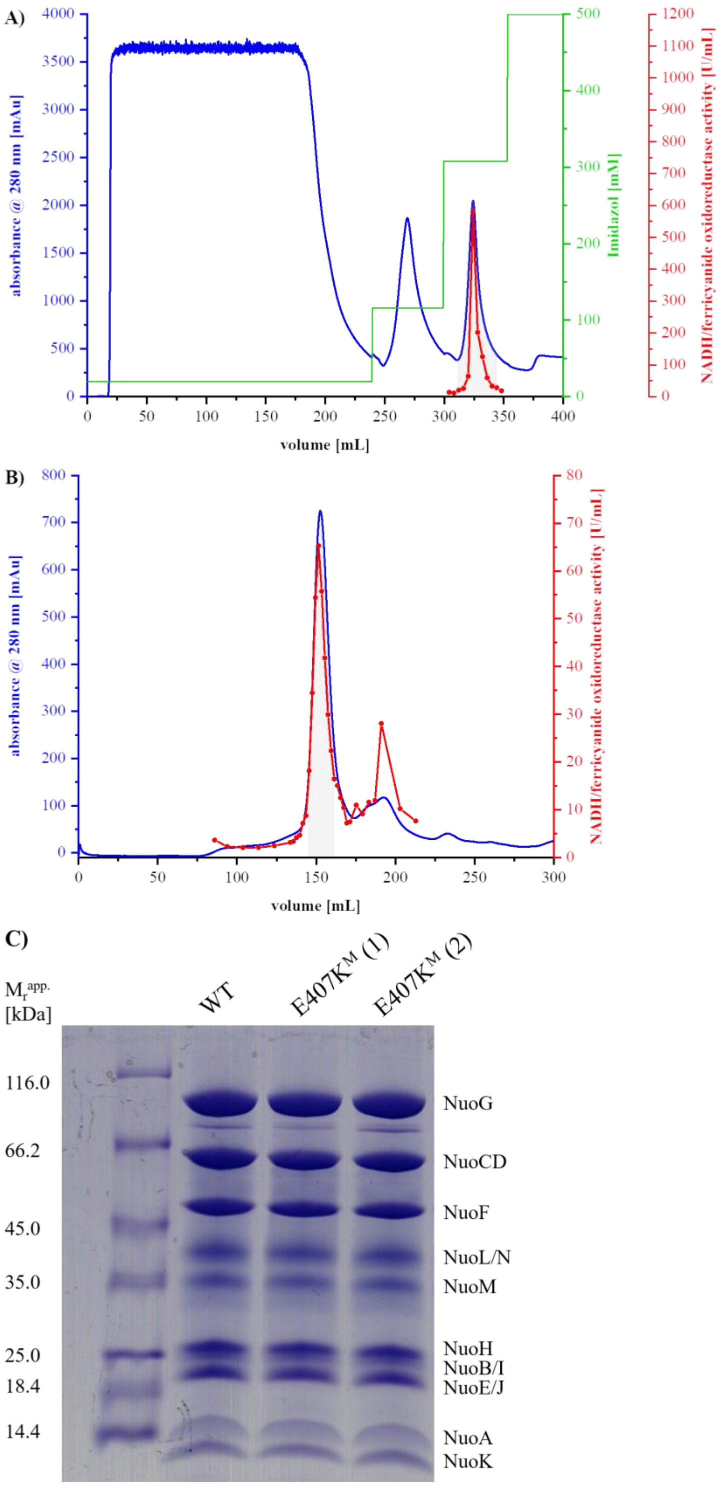

Fig. S3. Preparation and characterization of E407K^{M} variant. Elution profile of A) the affinity-chromatography on ProBond Ni^{2+}-IDA and B) the size exclusion chromatography on Superose 6. Peak fractions of activity (grey area) were used in the next step. Preparations of complex I and the variant showed virtually identical elution profiles. C) SDS-PAGE of both preparations revealing the presence of all complex I subunits. Two different preparations of E407K^{M} are shown. The faint band at around 80 kDa is a proteolytic digestion product of NuoG.

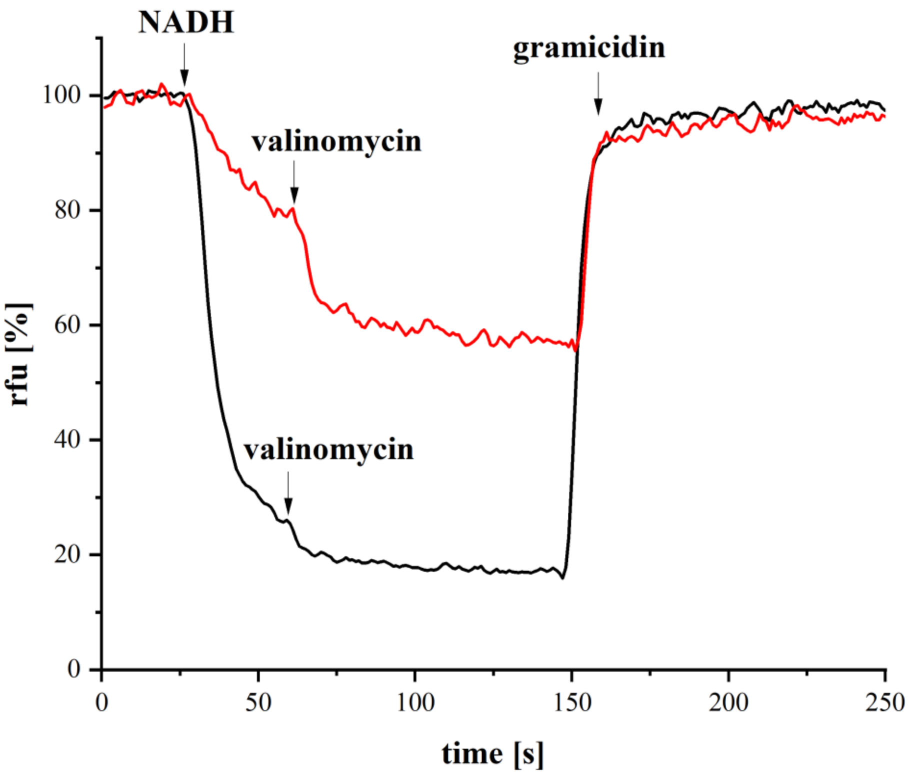

Fig. S4. Generation of ΔpH by *E. coli* complex I (black) and the E407K^{M} variant (red) reconstituted into liposomes measured as quench of the ACMA fluorescence. The reaction was started by addition of 130 µM NADH. Addition of 20 nM valinomycin led to a dissipation of ΔΨ and accelerated the reaction. The pH gradient was dissipated by an addition of gramicidin.

Table S1. Oligonucleotides used for site-directed mutagenesis. Newly generated codons are shown in bold, exchanged bases in italics. To proof the newly generated codons, silent mutations were inserted. The restriction sites are underlined. The ‘check’ oligonucleotides were used for DNA sequencing.

**Oligonucleotides**	**sequence 5’-3’**	**Restriction site**
nuoM_E407K_fwd	GCACCGGTAACTTCGTCGGC***AA*A**TTTATGATTCTGTTC GG<u>*A*AGCTT</u>CCAGGTTG	*HindIII*
nuoM_E407K_rev	CAACCTGGAAGCTTCCGAACAGAATCATAAATTTGCCG ACGAAGTTACCGGTGC	
Check_nuoM_E407K	GTATCCATACCCGCG	
